# Susceptibility to e-cigarette use and associated factors in high school youth, Oklahoma Youth Tobacco Survey, 2021–2022

**DOI:** 10.3389/fpubh.2024.1348926

**Published:** 2024-02-01

**Authors:** Shirley A. James, Ashley H. White, Fahad F. Kahn, Nasir Mushtaq, Sixia Chen, Laura A. Beebe

**Affiliations:** ^1^Department of Biostatistics and Epidemiology, Hudson College of Public Health, University of Oklahoma Health Sciences Center, Oklahoma City, OK, United States; ^2^Oklahoma State Department of Health, Oklahoma City, OK, United States

**Keywords:** susceptibility, electronic cigarettes (e-cigarettes), vaping, youth tobacco prevention, youth tobacco survey

## Abstract

**Introduction:**

Susceptibility predicts subsequent uptake of e-cigarettes (EC) by youth. This study identified factors associated with EC susceptibility among high school students who have never used a tobacco/nicotine product.

**Methods:**

The Oklahoma Youth Tobacco Survey was administered to a random sample of 36 Oklahoma High Schools during the 2021–2022 school year (*n* = 1,220 participating students). Associations between EC susceptibility and covariates were identified using stepwise logistic regression for weighted survey data.

**Results:**

More than one third of Oklahoma high school students who had never used tobacco or nicotine products (36.4%) were susceptible, and males had higher susceptibility than females (38.8 and 33.9%, respectively). In males, EC susceptibility was associated with race (Black, American Indian, and other were less susceptible), psychological distress (aOR = 2.4, 95% CI = 1.1, 4.8), disagreement that all tobacco products are dangerous (aOR = 3.1, 95% CI = 1.2, 7.9), and perception of little/no harm from secondhand vapor (aOR = 3.4, 95% CI = 2.1, 5.3). In females, identifying as gay, lesbian, or bisexual (aOR = 2.1, 95% CI = 1.1, 3.9), poor academic performance (aOR = 4.5, 95% CI = 1.6, 12.6), psychological distress (aOR = 2.6, 95% CI = 1.2, 5.5) and interacting with EC content on social media (aOR = 5.9, 95% CI = 1.9, 18.1) were associated with EC susceptibility.

**Conclusion:**

Males and females had different patterns of susceptibility to EC use. Understanding groups of adolescents most susceptible to using nicotine products can help target prevention efforts at home, in schools, and within communities.

## Background/Introduction

Electronic cigarette (EC) use among youth remains problematic and can lead to other forms of nicotine dependence, including smoking (1, 2). Previous research suggests adolescents who regularly used vaping products are up to four times more likely to have smoked in the past 30-days or to have initiated smoking (1). Similarly, there is a strong association between smoking initiation and regular vaping product use among youth (2).

Most adult tobacco use begins with tobacco experimentation during adolescence (1, 2). Of the wide array of tobacco products available, current high school (HS) students most often choose to experiment with ECs. In 2018, Gentzke and associates reported an adolescent 30-day EC prevalence of 27.7% using data from the National Youth Tobacco Survey (YTS) (3). This prevalence dropped to 19.6% in 2020 (4), 11.3% in 2021, and is currently 14.1% in 2022 (5, 6). While this drop in 30-day prevalence during the last 2 years is encouraging, EC use continues to be a concern, and a significant proportion of adolescents remain susceptible to initiation.

Several research studies have documented factors associated EC use in youth, including identifying as White, using other tobacco products, and having family members who use tobacco of any kind (7). Stress is also associated with both EC and tobacco use and can be related to school grades, peer pressure, gender diversity, and other stressors (7–10). Harm perception or the perception that ECs are less harmful and/or addictive than smoking traditional tobacco products is strongly associated with EC use among youth (8–10), as is exposure to EC advertisement and marketing. Alternatively, television, radio, and social media messaging exposing the dangers associated with tobacco use can increase the perception of harm and decrease susceptibility to tobacco initiation (11, 12).

Preventing initiation is an important step in averting nicotine dependence (1). Susceptibility precedes initiation of tobacco use of any kind (1). EC susceptibility is defined as a lack of firm, decisive, and robust denial of interest in initiating EC use among never users (1, 13, 14). Several studies have reported a strong association between EC susceptibility and initiation within youth (15–17).

A number of studies have evaluated susceptibility to EC use among adolescents, and findings vary based on sampling methods and measures. EC susceptibility has been associated with believing that ECs are less harmful than combustible tobacco products (18–20), believing that ECs are less addictive than combustible tobacco products (21), and having higher affluence (19, 22). Additional factors associated with EC susceptibility include being exposed to EC advertising (22), living in a household where members use ECs (18), and having family members or friends who smoke or vape (21, 22). Conversely, identifying as Black (18), Hispanic (18, 20), and female (18, 20) have been associated with a protective effect with regard to EC susceptibility. Studies limiting the analytic sample to youth who have never used any nicotine product are uncommon. The aim of this study was to determine variables associated with EC susceptibility among high school youth in Oklahoma who have never used any tobacco or nicotine product, including ECs.

## Methods

### Data

Data for this study were obtained from the Oklahoma Youth Tobacco Survey (OYTS), administered from November 2021 through May 2022. A multi-stage sampling design was used to draw the sample of students. The first stage involved selecting a random sample of public high schools. The second stage involved selecting three classes from each school, using simple random sampling without replacement. Finally, all students in each class were offered the opportunity to take the online survey. The OYTS included a final sample of 36 public high schools with a total sample size of 1,220 students. The analytic sample used in this study was students who never used any tobacco or nicotine product, and with complete information about grade level and age required for accurate weighting (*n* = 780).

### Outcome variable

Susceptibility to EC use was defined using the susceptibility index previously developed and validated for smoking susceptibility (1) and determined from the following four questions: “Have you ever been curious about using an e-cigarette?,” “Do you think you will try an e-cigarette soon?,” “Do you think you will use an e-cigarette in the next year?,” and “If one of your best friends were to offer you an e-cigarette, would you use it?” Possible answers included “definitely yes,” “probably yes,” “probably not,” and “definitely not.” Students were considered susceptible if they responded with any answer *except* “definitely not” to *any* of those questions.

### Measures

#### Demographic variables

Covariates included ethnicity, categorized as Hispanic or non-Hispanic; race, categorized as American Indian, Black, White, or other; grade level categorized as freshman/sophomore or junior/senior; and sex, categorized as male or female. Finally, students were asked if they spoke a language other than English in the home, with responses dichotomized as yes or no.

#### Sexual identity

When asked, “Which of the following best describes you?,” respondents self-identified into the following categories: straight; gay, lesbian, or bisexual; and unsure.

#### Grades in school

Respondents were asked, “During the last 12 months, how would you describe your grades in school?” Responses were coded as “As and Bs”; “C’s or lower”; and “graded on another scale or unsure.” Students graded on another scale were either on a pass/fail grading scale or using an individualized education plan for special education purposes.

#### Family affluence score

An affluence score was assigned based on four questions; “Does your family own a vehicle?” (no = 0, one = 1, and two or more = 2), “Do you have your own bedroom?” (no = 0 and yes = 1), “How many computers does your family own?” “(none = 0, one = 1, two = 2, and more than two = 3),” and “how many times in the last 12 months have you traveled on vacation with your family?” (Not at all = 0, once = 1, twice = 2, and more than twice = 3). Responses were summed with scores of five or less coded “low affluence,” and scores of six or more coded “high affluence,” consistent with prior studies (3–6).

#### Psychological distress

A psychological distress score was assigned based on four questions; “During the past 2 weeks, how often have you been bothered by having little interest or pleasure in doing things?,” “During the past 2 weeks, how often have you been bothered by feeling down, depressed, or hopeless?,” “During the past 2 weeks, how often have you been bothered by feeling nervous, anxious, or on edge?,” and “During the past 2 weeks, how often have you been bothered by feeling like you are not able to stop or control worrying?” Each question was coded not at all = 0, several days = 1, more than half of the days = 2, and nearly every day = 3. Consistent with prior literature, responses were summed with scores of five or less coded “none or low distress,” and scores six or more coded “moderate or severe” (3–6).

#### Harm perception

Four questions were used to determine EC harm perception. First, “How much do you think people harm themselves when they use ECs some days but not every day?” Responses of “No harm” or “a little harm” were combined and compared to “some harm” or “a lot of harm” combined. Next, responses to “Do you believe that ECs are (less addictive, equally addictive, or more addictive) than cigarettes?” were dichotomized as “equally/less/do not know” combined and compared to “more addictive.” Third, agreement with the statement “All tobacco products are dangerous” was assessed. Those who responded, “strongly agree” or “agree” were combined and compared to those who responded “disagree” or “strongly disagree.” Fourth, Do you think that breathing the vapor from other people’s EC causes “no harm,” “a little harm,” “some harm,” or “a lot of harm.” Respondents answering, “no harm” or “a little harm” were combined and compared to those who answered, “some harm” or “a lot of harm” (3–6).

#### Anti-tobacco messaging

Respondents were asked two questions about anti-tobacco messaging. Youth who responded yes to seeing or hearing The Real Cost ads in the past 12 months, and those selecting one or more anti-tobacco names or slogans they may have seen in the past 12 months were considered to have been exposed. Answers were summed and then dichotomized into 0 or 1 and 2 or more (3–6).

#### EC and tobacco product marketing

Exposure to EC and other tobacco marketing was assessed separately and from questions about four different sources: retail stores; internet; television, streaming services, or movies; and newspapers or magazines. Respondents were asked, “When you are using ‘each of these services’ how often do you see ads or promotions (for ECs; for cigarettes or tobacco products)?” Respondents could answer never, rarely, sometimes, most of the time, or always. They received one point for each answer of sometimes, most of the time, or always. Answers were summed and then dichotomized into 0 or 1 and 2 or more (2–4).

#### Social media

Among students responding they use social media, we captured social media exposure based on four questions. First, we asked “How often do you use social media?” Second, we asked “When you use social media, how often do you see posts of content related to e-cigarettes?” To assess interaction with social media, we then asked the following two questions: “When you use social media, how often do you post pictures of yourself or someone else using e-cigarettes?” and “When you use social media, how often have you liked, commented, or shared posts or content related to e-cigarettes?” We dichotomized each question separately, with those responding monthly or more frequently combined and compared to those responding, “less than monthly or never to these questions” (3–6). Those responding that they do not use social media were categorized in the “less than monthly or never” category.

### Statistical methods

Data were weighted to adjust for nonresponse and varying probabilities of selection with the underlying population of interest, with extreme weights trimmed. The weighting procedures included base weight, nonresponse adjustment, calibration, and trimming; done to incorporate sampling randomness, reduce nonresponse bias, and improve efficiency. Bivariate associations between covariates and the outcome variable, EC susceptibility, were examined using a Rai-Scott Chi-square test. Weighted multivariable logistic regression was conducted, analyzing the association between EC susceptibility and the series of independent variables using a stepwise selection procedure. Collinearity and interactions were examined in building the final model. Adjusted odds ratios were obtained for the association between EC susceptibility and independent variables. Respondents with missing outcome values were excluded from bivariate and multivariate analysis. Because there was an interaction with sex, all results are presented separately for males and females. All statistical analyses were conducted in SAS^®^ 9.4 (Carey, NC) with an alpha = 0.05. All statistical analyses incorporate design information including final weight, stratification, and clustering. The protocol was approved by Institutional Review Boards at both the Oklahoma State Department of Health (#21–12) and the University of Oklahoma Health Sciences Center (#13847).

## Results

Among students who had never used a tobacco or nicotine product, 24% self-identified as American Indian, 11% as Black, 60% as White, and 5% as a member of another race. Most students (82%) self-identified as being “straight” regarding sexual identity, and 78% reported earning A or B grades in school. A high percentage of female students were experiencing psychological distress compared to males (22.1% versus 8.4%). Most students (90%) agreed or strongly agreed that “all tobacco products are dangerous.” More than one-third (37%) responded that breathing vapor from other people’s ECs causes “little” or “no” harm. About half (48%) had seen two or more anti-tobacco advertisements in the past 12 months, and 92% were exposed to e-cigarette advertising in the past 12 months. Regarding social media, 41% of students had seen EC content on social media “monthly or more often,” while 8% had posted pictures, commented on, or shared posts about ECs (Table 1).

**Table 1:** Characteristics of high school students who have never used tobacco/nicotine products, by sex.

Variable	Total (*n* = 780)		Males (*n* = 404)		Females (*n* = 376)	
	Freq	Weighted % (95% CI)	Freq	Weighted % (95% CI)	Freq	Weighted % (95% CI)
Grade level						
Freshman-Sophomore	476	59.70 (49.98, 69.42)	249	59.46 (49.84, 69.09)	227	59.95 (46.09, 73.80)
Junior–Senior	304	40.30 (30.58, 50.02)	155	40.54 (30.91, 50.16)	149	40.05 (26.20, 53.91)
Race						
American Indian	166	23.91 (17.08, 30.75)	96	26.38 (16.88, 35.88)	70	21.31 (15.68, 26.94)
Black	75	11.41 (562, 17.21)	45	13.90 (6.12, 21.67)	30	8.81 (4.04, 13.57)
White	456	59.73 (52.01, 67.44)	222	54.80 (45.08, 64.52)	234	64.91 (57.70, 72.11)
Other	44	4.95 (1.72, 8.18)	23	4.92 (1.18, 8.67)	21	4.98 (1.60,8.35)
Ethnicity						
Hispanic	226	21.06 (10.25, 31.86)	113	19.83 (9.94, 29.72)	113	22.31 (9.84, 34.78)
Non-Hispanic	547	78.94 (68.14, 89.75)	287	80.17 (70.29, 90.06)	260	77.69 (65.22, 90.16)
Language other than English spoken at home						
Yes	207	23.71 (15.58, 31.84)	109	23.87 (17.34, 30.40)	98	23.54 (12.85, 34.24)
No	520	76.29 (68.16, 84.42)	265	76.13 (69.60, 82.67)	255	76.46 (65.76, 87.15)
Sexual identity						
Gay, lesbian or bisexual	67	8.84 (5.13, 12.54)	22	6.23 (2.01, 10.44)	45	11.51 (6.89, 16.13)
Straight	588	81.97 (78.16, 85.77)	319	85.17 (80.28, 90.07)	269	78.69 (74.83, 82.55)
Not sure	70	9.19 (6.78, 11.61)	33	8.60 (5.24, 11.95)	37	9.80 (7.30, 12.30)
Grades in school						
A’s and B’s	566	78.01 (70.24, 85.78)	270	70.20 (59.38, 81.02)	296	85.96 (79.96, 91.96)
C’s or lower	108	14.82 (9.81, 19.83)	70	20.48 (12.71, 28.24)	38	9.06 (4.19, 13.94)
Another scale/unsure	52	7.17 (3.79, 10.54)	35	9.32 (5.65, 12.99)	17	4.97 (1.22, 8.73)
Family affluence scale						
Low affluence	315	41.86 (34.50, 49.23)	177	47.01 (38.43, 55.59)	138	36.60 (27.81, 45.45)
High affluence	413	58.14 (50.77, 65.50)	198	52.99 (44.41, 61.57)	215	63.37 (54.55, 72.19)
Psychological distress (PHQ-4 scale)						
None or mild	637	84.69 (80.62, 88.77)	356	91.56 (88.65, 94.46)	281	77.61 (71.28, 83.93)
Moderate or severe	124	15.31 (11.23, 19.38)	39	8.44 (5.54, 11.35)	85	22.07 (16.07, 28.71)
Perception of harm when people use e-cigarettes some days but not every day						
Little/no harm	140	18.04 (14.51, 21.57)	81	20.89 (15.99, 25.80)	59	15.12 (9.62, 20.63)
Some/ a lot of harm	621	81.96 (78.43, 85.49)	311	79.11 (74.20, 84.01)	310	84.88 (79.37, 90.38)
Agreement with “all tobacco products are dangerous”						
Disagree/strongly disagree	77	9.74 (6.05, 13.42)	48	11.94 (6.42, 17.47)	29	7.48 (3.56, 11.40)
Agree/strongly agree	678	90.26 (86.58, 93.95)	341	88.06 (82.53, 93.58)	337	92.52 (88.60, 96.44)
Belief that e-cigarettes are less, equally, or more addictive than cigarettes						
Less, equally addictive, unsure	528	68.55 (64.95, 72.14)	279	70.83 (65.96, 75.69)	249	66.24(61.81, 70.67)
More addictive	231	31.45 (27.86, 35.05)	111	29.17 (24.31, 34.04)	120	33.76 (29.33, 38.19)
Belief about the harm from breathing the vapor from other people’s e-cigarettes						
Little or no harm	282	37.32 (32.53, 42.11)	149	38.35 (31.79, 44.92)	133	36.27 (28.84, 43.70)
Some or a lot of harm	473	62.68 (57.89, 67.47)	240	61.65 (55.08, 68.21)	233	63.73 (56.30, 71.16)
Anti-tobacco advertising seen in past 12 months						
0–1 ad	399	52.21 (44.24, 60.17)	196	48.49 (40.05, 56.93)	203	56.04 (46.58, 65.50)
2 or more	381	47.79 (39.83, 55.76)	208	51.51 (43.07, 59.60)	173	43.96 (34.50, 53.42)
E-cigarette advertising						
Not exposed	66	8.43 (6.21, 10.64)	39	10.53 (6.97, 14.10)	27	6.25 (2.95, 9.56)
Exposed	714	91.57 (89.36, 93.79)	365	89.47 (85.90, 93.03)	349	93.75 (90.44, 97.05)
Frequency of seeing e-cigarette-related content in social media posts						
Monthly or more often	312	41.44 (37.54, 45.34)	130	34.02 (2,887, 39.16)	182	49.09 (43.03, 55.16)
Never or less than monthly	468	58.56 (54.66, 62.46)	274	65.98 (60.84, 71.13)	194	50.91 (44.84, 56.97)
Frequency of posting pictures of self or someone else using e-cigarettes, or liking, commenting on, or sharing posts related to e-cigarettes on social media						
Monthly or more often	71	8.44 (5.79, 11.09)	36	8.29 (4.92, 11.67)	35	8.60 (5.52, 11.68)
Never or less than monthly	709	91.56 (88.91, 94.21)	368	91.71 (88.33, 95.08)	341	91.40 (88.33, 94.48)

Oklahoma Youth Tobacco Survey 2021–2022 (*n* = 780).

Overall, 36% of students were susceptible to EC use: 39% of males and 34% of females. In males, susceptibility to EC use was higher among White students (44%) than Black (24%), or American Indian (36%) students. A higher proportion of students who self-identified as gay, lesbian, or bisexual (53%) were susceptible to EC use compared to those who self-identified as “straight” (34%). A larger proportion of students earning “C” grades or less were susceptible to EC use (52%) compared to those earning grades of “A” and “B” (34%) grades. More than half of students reporting high levels of psychological distress (53%) were susceptible to EC use compared to those reporting mild or no stress (34%). Overall, a large percentage of students who disagreed or strongly disagreed with the statement that “all tobacco” products are dangerous (51%) were susceptible to EC use compared to those who agreed or strongly agreed (34%). While almost half of all students who thought that “breathing vapor” from other people’s vaping products causes “little” or “no harm” were susceptible (46%), susceptibility was higher in males (52%), compared to females (39%). Of students who posted pictures of themselves or someone else using vaping products on social media, or who commented on, or shared posts related to ECs monthly or more often, 62% were susceptible overall (65% of males and 58% of females) (Table 2).

**Table 2:** E-cigarette susceptibility among high school students who never used tobacco/nicotine products by sex and variables of interest.

	Overall			Males			Females		
Variable	*n* = 780	Weighted % and 95 CI	*p*-value	*n* = 404	Weighted % and 95 CI	*p*-value	*n* = 376	Weighted % and 95 CI	*p*-value
E-cigarette susceptibility									
Susceptible	303	36.39 (32.89, 39.88)	**<0.0001**	159	38.76 (35.10, 42.42)	**<0.0001**	144	33.94 (29.03, 38.86)	**<0.0001**
Not susceptible	477	63.61 (60.12, 67.11)		245	61.24 (57.58, 64.90)		232	66.06 (61.15, 70.97)	
Grade level									
Freshman-Sophomore	189	37.66 (31.20, 44.12)	0.5629	101	41.00 (35.75, 46.24)	0.2306	88	34.25 (24.47, 44.03)	0.9300
Junior–Senior	114	34.50 (27.93, 41.17)		58	35.47 (28.83, 42.11)		56	33.48 (22.65, 44.31)	
Race									
American Indian	66	36.89 (28.60, 45.18)	0.2350	34	35.95 (26.06, 45.84)	0.0651	32	38.11 (24.05, 52.17)	0.5368
Black	25	26.21 (17.40, 35.01)		14	24.43 (13.14, 35.72)		11	29.16 (12.98, 45.34)	
White	176	37.35 (31.30, 43.39)		95	44.06 (35.76, 52.35)		81	31.39 (23.41, 39.37)	
Other	18	40.33 (29.05, 51.60)		9	35.49 (21.28, 49.70)		9	45.35 (23.43, 67.28)	
Ethnicity									
Hispanic	99	43.74 (38.97, 48.51)	**0.0163**	51	46.27 (36.29, 56.25)	0.1858	48	41.45 (32.55, 50.36)	0.0913
Non-Hispanic	203	34.87 (30.28, 39.46)		108	37.73 (32.42, 43.05)		95	31.86 (25.65, 38.08)	
Language other than English spoken at home									
Yes	90	41.01 (32.81, 49.21)	0.2182	48	42.84 (30.00, 55.68)	0.4313	42	39.13 (29.99, 48.26)	0.2408
No	190	34.64 (29.33, 39.95)		96	36.61 (30.48, 42.75)		94	32.66 (26.06, 39.25)	
Sexual identity									
Gay, lesbian or bisexual	37	52.71 (39.88, 64.54)	**0.0209**	12	50.68 (31.32, 70.05)	0.3513	25	53.83 (35.07, 72.58)	**0.0087**
Straight	210	33.59 (28.83, 38.35)		120	37.76 (32.76, 42.76)		90	28.97 (22.44, 35.50)	
Not sure	32	41.95 (27.33, 56.57)		12	31.71 (14.27, 49.16)		20	51.13 (31.12, 71.15)	
Grades in school									
A’s and B’s	209	33.72 (29.80, 37.64)	**0.0034**	105	38.54 (32.56, 44.51)	**0.0351**	104	29.71 (25.38, 34.05)	**0.0007**
C’s or lower	57	52.02 (38.73, 65.31)		35	47.16 (34.07, 60.25)		22	63.19 (41.19, 85.20)	
Another scale/unsure	14	30.55 (19.51, 41.59)		6	18.40 (6.40, 30.40)		8	53.73 (30.56, 76.90)	
Family affluence scale									
Low affluence	127	37.16 (32.19, 42.13)	0.6470	69	38.65 (32.43, 44.87)	0.9113	58	35.22 (26.54, 43.90)	0.7386
High affluence	155	35.62 (29.94, 41.30)		77	38.02 (30.19, 45.84)		78	33.58 (27.10, 40.05)	
Psychological distress									
None or mild	230	33.54 (29.41, 37.67)	**0.0030**	132	37.51 (33.60, 41.42)	0.0564	98	28.71 (22.28, 35.13)	**0.0217**
Moderate or severe	65	52.71 (42.39, 63.02)		23	54.36 (38.01, 70.72)		42	52.06 (35.30, 68.83)	
Perception of how much harm people cause themselves when they use e-cigarettes some days but not every day									
Little/no harm	69	49.07 (37.62, 60.52)	0.0191	40	52.41 (40.67, 64.14)	**0.0105**	29	44.36 (26.07, 62.65)	0.1993
Some/a lot of harm	225	33.22 (28.73, 37.72)		113	34.67 (30.21, 39.14)		112	31.84 (26.00, 37.68)	
Agreement with “All tobacco products are dangerous”									
Disagree/strongly disagree	40	51.22 (43.15, 59.28)	**0.0042**	27	55.59 (36.72, 74.46)	0.0849	13	44.10 (18.57, 69.63)	0.3467
Agree/strongly agree	250	34.14 (29.51, 38.78)		124	35.85 (30.08, 41.62)		126	32.48 (27.15, 37.81)	
Belief that e-cigarettes are less, equally, or more addictive than cigarettes									
Less, equally, unsure	201	36.05 (31.66, 40.44)	0.08912	107	38.36 (34.97, 41.75)	0.7787	94	33.56 (26.63, 40.48)	0.9377
More addictive	93	36.61 (29.24, 43.99)		46	39.51 (30.66, 48.34)		47	34.08 (23.50, 44.67)	
Belief about the harm from breathing the vapor from other people’s e-cigarettes									
Little or no harm	135	45.59 (38.74, 52.43)	**0.0003**	76	51.57 (44.36, 58.79)	**0.0003**	59	39.14 (27.53, 50.76)	0.2147
Some or a lot of harm	156	30.28 (26.33, 34.23)		76	30.20 (24.72, 35.69)		80	30.36 (23.98, 36.74)	
Anti-tobacco advertising seen in past 12 months									
0–1 ad	145	33.54 (29.10, 37.99)	0.1118	77	38.88 (33.33, 44.43)	0.9625	68	28.79 (22.48, 35.09)	**0.0487**
2 or more	158	39.49 (33.71, 45.27)		82	38.64 (31.66, 45.63)		76	40.51 (31.49, 49.53)	
E-cigarette advertising									
Not exposed	20	32.57 (20.10, 45.04)	0.5644	13	37.79 (20.82, 54.76)	0.9050	7	23.52 (3.97, 43.06)	0.3296
Exposed	283	36.74 (32.60, 40.87)		146	38.87 (34.64, 43.10)		137	34.64 (29.30, 39.98)	
Frequency of seeing e-cigarette-related content in social media posts									
Monthly or more often	142	42.48 (36.60, 48.37)	0.0154	58	43.35 (34.58, 52.11)	0.2173	84	41.87 (31.74, 51.99)	0.0663
Never or less than monthly	161	32.07 (27.18, 36.96)		101	36.39 (31.30, 41.48)		60	26.30 (17.28, 35.32)	
Frequency of posting pictures of self or someone else using e-cigarettes, or liking, commenting on, or sharing posts related to e-cigarettes on social media									
Monthly or more often	42	61.57 (50.18, 72.95)	**0.0002**	21	65.31 (47.07, 83.55)	**0.0088**	21	57.84 (37.41, 78.27)	**0.0129**
Never or less than monthly	261	34.06 (30.23, 37.90)		138	36.36 (32.09, 40.62)		123	31.69 (26.85, 36.54)	

Oklahoma Youth Tobacco Survey, 2021–2022 (*n* = 780). Bolded values indicate statistical significance at the 0.05 level or below.

### Multivariate analysis results for males and females

#### Males

When compared to white male students, after adjusting for other variables in the model, the odds of susceptibility to EC use in American Indian and Black male students were lower (aOR = 0.46, 95% CI = 0.23, 0.90 and 0.44, 95% CI = 0.20, 0.96, respectively). In male students, the odds of EC susceptibility were also considerably lower among those who were graded on a different grading scale (aOR = 0.31 with 95% CI = 0.14, 0.70) compared to those who made “A” or “B” grades. After adjusting for other variables in the model, the odds of EC susceptibility in male students who reported moderate or severe levels of psychological stress were more than twice as high as for those reporting mild or no stress (aOR = 2.35, 95% CI = 1.14, 4.81). Likewise, the odds of EC susceptibility among male students who disagreed or strongly disagreed with the statement “all tobacco products are dangerous” were three times higher (aOR = 3.07, 95% CI = 1.19, 7.92) compared to those who agreed or strongly agreed. The odds EC susceptibility among those who perceived little or no harm from breathing vapor from other people’s ECs were more than three times higher when compared to those who perceived some or a lot of harm (aOR = 3.35 with 95%CI = 2.12, 5.30) (Table 3).

**Table 3:** Factors associated with e-cigarette susceptibility, by sex, crude, and adjusted odds ratios with 95% CIs.

	Males*		Females**	
Variable	Crude odds ratio (95% CI)	Adj odds ratio (95% CI)	Crude odds ratio (95% CI)	Adj odds ratio (95% CI)
Grade level				
Freshman-Sophomore	1.26 (0.85, 1.89)		1.04 (0.46, 2.34)	
Junior–Senior	Referent		Referent	
Race				
American Indian	0.71 (0.39, 1.31)	**0.46 (0.23, 0.90)**	1.35 (0.64, 2.83)	1.75 (0.92, 3.31)
Black	**0.41 (0.19, 0.90)**	**0.44 (0.20, 0.96)**	0.90 (0.37, 2.17)	0.88 (0.34, 2.31)
White	Referent		Referent	
Other	0.70 (0.37, 1.31)	**0.36 (0.16, 0.81)**	1.81 (0.67, 4.88)	2.58 (0.57, 11.76)
Ethnicity				
Hispanic	1.42 (0.82, 2.48)		1.51 (0.92, 2.50)	
Non-Hispanic	Referent		Referent	
Language other than English spoken at home				
Yes	1.30 (0.64, 2.63)		1.33 (0.79, 2.22)	
No				
Sexual identity				
Gay, lesbian or bisexual	1.69 (0.71, 4.03)		**2.86 (1.34, 6.10)**	**2.10 (1.13, 3.90)**
Straight	Referent		Referent	
Not sure	0.77 (0.31, 1.91)		2.57 (0.96, 6.83)	**4.02 (1.30, 12.38)**
Grades in school				
A’s and B’s	Referent		Referent	
C’s or lower	1.42 (0.71, 2.86)	1.65 (0.90, 3.05)	**4.06 (1.65, 9.99)**	**4.50 (1.61, 12.56)**
Another scale/unsure	**0.36 (0.15, 0.87)**	**0.31 (0.14, 0.70)**	**2.75 (1.06, 7.11)**	2.60 (0.79, 8.57)
Family affluence scale				
Low affluence	Referent		Referent	
High affluence	0.97 (0.59, 1.61)		0.93 (0.59, 1.47)	1.67 (0.97, 2.90)
Psychological distress				
None or mild	Referent		Referent	
Moderate or severe	1.99 (0.97, 4.07)	**2.35 (1.14, 4.81)**	2.35 (1.14, 4.81)	**2.58 (1.21, 5.53)**
Perception of harm when people use e-cigarettes some days but not every day				
Little/no harm	**2.08 (1.22, 3.52)**		1.71 (0.72, 4.08)	1.80 (0.88, 3.68)
Some/ a lot of harm	Referent		Referent	
Agreement with “All tobacco products are dangerous”				
Disagree/strongly disagree	2.24 (0.87, 5.76)	**3.07 (1.19, 7.92)**	1.64 (0.55, 4.88)	
Agree/strongly agree	Referent		Referent	
Belief that ECs are less, equally, or more addictive than cigarettes				
Equally, less/do not know	0.95 (0.67, 1.36)		0.98 (0.52, 1.83)	
More addictive	referent		referent	
Belief about the harm from breathing the vapor from other people’s e-cigarettes				
Little or no harm	**2.46 (1.63, 3.72)**	**3.35 (2.12, 5.30)**	1.48 (0.78, 2.81)	
Some or a lot of harm	Referent		Referent	
Anti-tobacco advertising seen in past 12 months				
0–1 ad	1.01 (0.65, 1.56)		**0.59 (0.36, 0.99)**	
2 or more	Referent		Referent	
E-cigarette advertising				
Not exposed	Referent		Referent	
Exposed	1.05 (0.47, 2.32)		1.72 (0.56, 5.34)	
Frequency of seeing e-cigarette-related content in social media posts				
Monthly or more	1.34 (0.83, 2.16)		2.02 (0.96, 4.26)	
Never or < monthly	Referent		Referent	
Frequency of posting pictures of self or someone else using e-cigarettes, or liking, commenting on, or sharing posts related to e-cigarettes on social media				
Monthly or more	**3.30 (1.34, 8.08)**		**2.96 (1.21, 7.22)**	**5.91 (1.94, 18.10)**
Never or < monthly	Referent		Referent	

Oklahoma Youth Tobacco Survey, 2021–2022. *Male odds ratios were adjusted for variables retained in the stepwise logistic model: race, grades in school, psychological distress, perceived danger of tobacco products, and vapor harm perception. **Female odds ratios were adjusted for variables retained in the stepwise logistic model: race, sexual identity, grades in school, psychological distress, and social media interaction. Bolded values indicate statistical significance at the 0.05 level or below.

#### Females

After adjusting for other variables in the model, the odds of EC susceptibility among females self-identifying as gay, lesbian, or bisexual were two times higher (aOR = 2.10, 95% CI = 1.13, 3.90), and for those who were unsure of their sexual identity, the odds were four times higher (aOR = 4.02, 95% CI = 1.30, 12.38) compared to those who self-identified as “straight.” The odds of susceptibility among female students who made “C” grades or lower were more than four times higher than for those making “A” or “B” grades (aOR = 4.50, 95% CI = 1.61, 12.56) and were almost three times higher for those under moderate or severe psychological stress compared to those with mild or no stress (aOR = 2.58, 95% CI = 1.21, 5.53). The odds of susceptibility to EC use in female students who interacted about EC use on social media were almost six times higher than for those who did not (aOR = 5.91, 95%CI = 1.94, 18.10) (Table 3).

## Discussion

More than one third of HS students who never used tobacco products were found to be susceptible to EC use. Patterns of susceptibility differed between male and female students. White males were more likely to be susceptible than Black or American Indian males. As reported by others (18, 20), this study found an association between identifying as White and EC susceptibility; however, in our study this only occurred with male students. Male students with low levels of EC/tobacco harm perception were more likely to be susceptible to EC initiation. Females, however, demonstrated an association between susceptibility and both psychological stress, as well as poorer academic performance. Females who interacted in social media about EC products were also more likely to be susceptible to EC initiation. Understanding these differences can assist with focused and evidence-based tobacco/nicotine prevention measures. An important step in tobacco prevention is averting tobacco initiation and susceptibility among youth, especially with popular tobacco products like ECs (1, 2).

Amrock and associates reported a study suggesting that adolescents cannot accurately assess the potential danger of ECs. They noted those who believe ECs are less harmful than combustible tobacco products are more likely to initiate their use (9). In our study, harm perception was only associated with EC susceptibility in male students and in only two of the four harm perception questions, agreeing that “all tobacco products are dangerous,” and that “breathing vapor from other’s ECs causes some or a lot of harm.” Because other authors have reported associations between harm perception and 30-day vaping prevalence (7, 17, 23, 24), continued public health education efforts are warranted. Previous research has reported that heightened harm perception is associated with lower EC susceptibility (with odds ratios between 0.60 and 0.23) (20, 21), while lower levels of harm perception have been associated with increases EC susceptibility (with odds ratios ranging from 2.2 to 4.9) (18).

Students experience a wide variety of stressors during their high school years. In this study, a higher percentage of female students demonstrated psychological distress, which in turn was associated with a higher level of susceptibility to EC use, after controlling for other covariates. Both male and female students experiencing distress had a higher prevalence of susceptibility. Female students demonstrated an association between grades earned in school and susceptibility; and had a higher odds of EC susceptibility when their grades dropped lower than a “B” level. Interestingly, Jha and associates found youth who needed stress relief were more likely to use ECs (13). However, the youth who attempted EC use as a form of stress relief reported higher stress levels after use. Research suggests EC prevention strategies for high school students should focus on stress reduction and healthy coping strategies (9, 22, 25).

While exposure to EC advertising on social media was *not* associated with EC susceptibility in either male or female students, *active interaction* on social media sites *was*. In female students, posting pictures, making comments about, or interacting with others about EC use was highly associated with EC susceptibility. A similar finding was reported by Vogel and associates, who found students who *engaged* in social media on a regular basis demonstrated higher intent to use ECs, along with a lower perception of the danger of EC use (26). This finding warrants further investigation about the potential success of monitoring social media sites in youth at risk for tobacco use, and providing intervention before initiation occurs.

This study adds information not yet published about differences in susceptibility in male and female adolescent students. Because this study was conducted with students who had never used any type of nicotine or tobacco product, these results are also unique. Limitations of the current study warrant discussion. This is a cross sectional study, and as such, causal inferences are not valid. While this study involved youth in Oklahoma, the sample of high school students never using nicotine and tobacco products was relatively small (*n* = 780) and from a single state; thus, generalizability may be limited. Sample sizes for several sub-groups of interest in this study were small, specifically those involving racial and sexual minority groups. Although weighting procedures intend to account for non-response, the overall response rate of schools and classrooms was less than optimal (44%). Finally, all estimates are based on self-reported data, which might be affected by information bias. As is typical with most surveys, data for all factors likely to be associated with susceptibility were not included.

Understanding EC susceptibility can assist with focused and evidence-based tobacco/nicotine prevention measures. An important step in tobacco prevention is averting tobacco initiation and susceptibility among youth.

## Data availability statement

The raw data supporting the conclusions of this article will be made available by the authors, without undue reservation.

## Ethics statement

The studies involving humans were approved by the University of Oklahoma Health Sciences Center. The studies were conducted in accordance with the local legislation and institutional requirements. Written informed consent for participation in this study was provided by the participants’ legal guardians/next of kin. Written informed consent was obtained from the individual(s) for the publication of any potentially identifiable images or data included in this article.

## Author contributions

SJ: Conceptualization, Formal analysis, Investigation, Methodology, Writing – original draft. AW: Formal analysis, Investigation, Writing – review & editing. FK: Funding acquisition, Supervision, Validation, Writing – review & editing. NM: Formal analysis, Investigation, Supervision, Writing – review & editing. SC: Formal analysis, Software, Supervision, Writing – review & editing. LB: Conceptualization, Formal analysis, Investigation, Methodology, Project administration, Supervision, Validation, Visualization, Writing – original draft.
